# The status of evolutionary medicine education in North American medical schools

**DOI:** 10.1186/s12909-015-0322-5

**Published:** 2015-03-08

**Authors:** Brandon H Hidaka, Anila Asghar, C Athena Aktipis, Randolph M Nesse, Terry M Wolpaw, Nicole K Skursky, Katelyn J Bennett, Matthew W Beyrouty, Mark D Schwartz

**Affiliations:** 1 University of Kansas Medical Center, 3901 W Rainbow Blvd, Kansas City, KS 66160 USA; 2 McGill University, 3700 McTavish Street, Room 244, Montréal, Québec H3A 1Y2 Canada; 3 Arizona State University, 411 North Central Avenue, Phoenix, AZ 85004 USA; 4 Penn State College of Medicine, 500 University Drive, Hershey, PA 17033 USA; 5 New York University School of Medicine, 550 1st Ave, New York, NY 10016 USA

**Keywords:** Evolutionary biology, Evolutionary medicine, Medical education, Medical school curriculum, Curriculum dean, Survey

## Abstract

**Background:**

Medical and public health scientists are using evolution to devise new strategies to solve major health problems. But based on a 2003 survey, medical curricula may not adequately prepare physicians to evaluate and extend these advances. This study assessed the change in coverage of evolution in North American medical schools since 2003 and identified opportunities for enriching medical education.

**Methods:**

In 2013, curriculum deans for all North American medical schools were invited to rate curricular coverage and perceived importance of 12 core principles, the extent of anticipated controversy from adding evolution, and the usefulness of 13 teaching resources. Differences between schools were assessed by Pearson’s chi-square test, Student’s t-test, and Spearman’s correlation. Open-ended questions sought insight into perceived barriers and benefits.

**Results:**

Despite repeated follow-up, 60 schools (39%) responded to the survey. There was no evidence of sample bias. The three evolutionary principles rated most important were antibiotic resistance, environmental mismatch, and somatic selection in cancer. While importance and coverage of principles were correlated (r = 0.76, *P* < 0.01), coverage (at least moderate) lagged behind importance (at least moderate) by an average of 21% (SD = 6%). Compared to 2003, a range of evolutionary principles were covered by 4 to 74% more schools. Nearly half (48%) of responders anticipated igniting controversy at their medical school if they added evolution to their curriculum. The teaching resources ranked most useful were model test questions and answers, case studies, and model curricula for existing courses/rotations. Limited resources (faculty expertise) were cited as the major barrier to adding more evolution, but benefits included a deeper understanding and improved patient care.

**Conclusion:**

North American medical schools have increased the evolution content in their curricula over the past decade. However, coverage is not commensurate with importance. At a few medical schools, anticipated controversy impedes teaching more evolution. Efforts to improve evolution education in medical schools should be directed toward boosting faculty expertise and crafting resources that can be easily integrated into existing curricula.

**Electronic supplementary material:**

The online version of this article (doi:10.1186/s12909-015-0322-5) contains supplementary material, which is available to authorized users.

## Background

Evolutionary biology is emerging as a useful tool for insight into major medical and public health challenges of the 21^st^ century [1-3]. The evolutionary perspective of cancer initiation and progression as an ecologic process of clonal selection [4] has led cancer researchers to treatment approaches for advanced disease aimed at controlling the replication of the most malignant cells rather than total eradication [5,6]. In the study of autoimmune disease, epidemiological studies have revealed increased risk from the loss of co-evolved parasites [7]; intestinal worms (helminths) are being tested as treatments for allergic rhinitis [8], inflammatory bowel disease [9], and multiple sclerosis [10]. In antibiotic treatment, providers are recognizing how medical practice influences pathogen evolution and are implementing antibiotic stewardship programs [11] to combat rising resistance to antibiotics [12,13]. Metabolic disorders and nutrition are increasingly being understood in a historical perspective that considers the mismatch between our genes and modern environment [14,15] which helps to explain why diabetes and obesity have become global health crises [16] and may have important implications for clinical management of type 2 diabetes [17]. These are just some of the ways that evolutionary principles are being used to better understand and manage human health [18,19].

The current study investigates the extent to which medical curricula provide physicians with the evolutionary knowledge needed to apply and extend these advances, while identifying obstacles to and opportunities for enhancing medical education. Understanding evolution deepens a physician’s understanding. As the scientific foundation of modern biology, evolution provides a conceptual framework that connects and organizes the often fragmented mountains of minutiae that medical students must memorize [20]. Understanding evolution allows physicians to view the human body not as an engineered machine, but a network of compromises. It also encourages asking, investigating, and answering *why?* questions about vulnerability to disease [21,22]. Why does a gene for a lethal disease (e.g., sickle cell anemia) exhibit a high prevalence in some human populations [23]? Why is asthma more common now [24]? Why does fetal undernourishment lead to an increased risk of adult metabolic disease [25]? Why do we age [26,27]? Physicians who learn the principles of evolutionary biology are in a better position to tackle these and other fundamental questions in medicine and public health.

Previous work evaluating the status of evolutionary biology in medical education has been limited [3,28-30]. In a 2001 study of medical schools in the U.K., 37% of the heads of medical schools reported covering evolution [28]. In a 2003 survey of North American medical school deans, 48% agreed that evolutionary biology is important knowledge for physicians [29], but they reported sparse coverage of core evolutionary concepts: 20% of deans reported 0 hours devoted to teaching evolutionary biology [29]. Here, we follow up that 2003 study with a revised survey of North American medical school deans intended to (a) measure current coverage of core topics in evolutionary medicine in curricula; (b) identify factors that are associated with coverage; and (c) elicit from curriculum deans the teaching resources that would be most useful to address the gaps in evolution education in medical schools.

## Methods

### Study subjects

Survey packets were mailed to curriculum deans at all 153 North American schools that award either a doctor of medicine or doctor of osteopathic medicine degree. Recipients were identified by information on institutional websites and clarified by telephone if needed. McGill University’s Ethics Review Board approved all aspects of the study on September 25th, 2012 (File # 52–0712). Confidentiality of respondents was promised and protected.

### Survey administration and questionnaire

After receiving an email alerting them to the survey, participants were sent envelopes via certified mail containing a cover letter that included support from the Institute of Medicine, a 36-item questionnaire (Additional file 1), and a stamped addressed return envelope. Non-respondents were sent a replacement packet and then up to four follow-up emails with a link to an electronic version of the questionnaire, then a follow-up phone call.

Deans were asked to answer questions about the composition of their faculty; the quantity of curricular time devoted to teaching evolution; the priority of training future researchers versus clinicians; the value placed on an undergraduate course in evolutionary biology; the presence of an evolutionary medicine student club; and potential controversy from adding more evolutionary content. Controversy was queried as “Would adding more content about evolution to the curriculum at your school arouse controversy among students, faculty, legislators, or donors?”

The primary outcome was current teaching (coverage) of 12 core evolutionary medicine topics in their school’s curriculum rated as: *not covered*, *covered briefly*, *covered moderately*, or *covered in depth.* The importance of teaching each of these 12 topics was also rated: *not important*, *minor importance*, *moderate importance*, or *essential*. We calculated the mean rating of overall curricular coverage and importance (ranging from 1 to 4, least to most) for each school. The topics were chosen from a 2010 article about how to make evolutionary biology a basic science for medicine [1]: maladaptive anatomy; defenses versus defects; pathogen virulence; proximate versus evolutionary explanation; somatic selection; trade-offs; environmental mismatch; hygiene hypothesis; senescence; sexual selection; antibiotic resistance; and levels of selection. Two curriculum deans checked style to ensure the wording was accessible to medical education leaders. Each topic was based on an evolutionary principle and illustrated with a learning objective. The instructions for these items and an example question are below:

- Each of the items below consists of a topic, a related evolutionary principle, and an example of a learning objective. Please indicate your personal assessment of the importance of teaching the topic/principle, and the amount of coverage of the topic in the current curriculum at your school by circling one response for each question.
- *How natural selection shapes defenses:* Many medical complaints are about averse but protective defenses such as pain, cough, vomiting, diarrhea, and anxiety. Explain how to distinguish symptoms that are defenses from those that arise from bodily defects, and the importance of making this distinction.

Schools also rated 13 potential teaching resources as: *not useful*, *possibly useful*, *likely useful*, or *definitely useful*. The survey concluded with open-ended questions about the utility of evolutionary principles in medicine, why evolutionary biology has not been fully applied in medicine, and suggestions for better incorporating evolution into medical education.

General characteristics of each school were determined by the survey (e.g., priority of training future researchers) or Internet searches of each institution (location, state voting record – Democrat versus Republican – from the 2012 presidential election, public versus private, and ranking quartile by National Institutes of Health funding).

### Comparison of 2003 and 2013 survey responses

We compared responses to identical items from the 2003 and 2013 studies to assess change in these variables. These comparisons were limited to medical schools (a) with evolutionary biology faculty, (b) reporting that they taught certain evolutionary topics, and (c) indicating that teaching evolutionary topics would raise controversy. In the previous survey, the coverage of topics was queried by a yes/no response to a topic without an explanation of what the term meant. Although the schools participating in the two surveys were not identical, the differences in responses to these items provided insight into national trends.

### Statistical analysis

All analyses were performed using JMP version 9.0.2 (SAS Institute Inc., Cary, NC). Global coverage among groups was compared by t-test or analysis of variance. Characteristics of responding schools were compared with the total population of 153 using chi-squared tests. Correlations between mean curricular coverage of evolution topics and factors potentially influencing coverage were assessed by Spearman’s rank correlation coefficient. Potential influences included the mean importance of the topics, curriculum hours devoted to evolution topics, curriculum hours devoted to teaching how to apply these topics to medical problems, number of faculty with a Ph.D. in evolution, number of faculty whose research is based in evolutionary biology, presence of a student club or interest group in evolutionary medicine, anticipated controversy of adding evolution content to the curriculum, and general medical school characteristics. Acceptable Type I error (α) was set at 0.05 without adjustment for multiple comparisons.

### Qualitative response coding

Three reviewers independently read all responses to the open-ended questions. Reviewers met and formed a consensus on common themes and categories (sub-themes). After agreeing on a coding scheme, reviewers independently coded all data into themes and categories. Reviewers reconvened to discuss discrepancies and unanimously classify responses. Quotes that illustrated themes and categories were selected.

## Results

### Study participants

Sixty (39%) medical school curriculum deans responded to the survey. The responding medical schools’ general characteristics are described in Table 1, compared to the full sample of 153 schools in North America. There was no evidence that the sample differed significantly from the population of medical schools on geography, public or private, or political environment. There were several incomplete responses. All percentages were calculated from the number of schools that responded to each question and the denominator is presented when it is not 60.

**Table 1:** **Characteristics of responding medical schools compared to the population of North American medical schools in 2013**

Characteristics		Sample	Population	*P* ^ a ^
		N = 60	N = 153	
		n (%)	n (%)	
Location	United States total	54 (90%)	139 (91%)	0.82
	Midwest	15 (28%)	33 (24%)	0.87
	Northeast	13 (24%)	31 (22%)	
	South	20 (37%)	56 (40%)	
	West	6 (11%)	19 (14%)	
	State voted Democrat in last presidential election	35 (65%)	85 (61%)	0.57
	State voted Republican in last presidential election	19 (35%)	54 (39%)	
	Canada total	6 (10%)	14 (9%)	0.82
	Atlantic	1 (17%)	2 (12%)	0.34
	Central	1 (17%)	8 (59%)	
	Prairie Province	3 (50%)	3 (24%)	
	West Coast	1 (17%)	1 (7%)	
Public or Private	Public	37 (62%)	86 (56%)	0.48
	Private	23 (38%)	67 (44%)	
Ranking	Not published	16 (27%)	69 (45%)	0.08
	1st Quartile	10 (17%)	21 (14%)	
	2nd Quartile	10 (17%)	21 (14%)	
	3rd Quartile	8 (13%)	21 (14%)	
	4th Quartile	16 (27%)	21 (14%)	
Priority of training future researchers is…	Low	9 (15%)	n/a	n/a
	Medium	26 (43%)		
	High	17 (28%)		
	Very high	8 (13%)		

^a^*P*-values comparing characteristics of study sample with those of the complete population of medical schools were calculated using Pearson’s chi-square test.

### School characteristics related to evolutionary biology

Forty percent (18/45) valued an undergraduate course in evolutionary biology for admission, but a course in evolution was not required by any school and was recommended by only 2 (4%). Two schools (4%; 2/53) had an evolutionary medicine student club or interest group. Forty-eight schools responded to whether adding evolution content to the curriculum would arouse controversy, 25 schools (52%) responded no; 11 (23%) said yes, but it would not pose any problems; 7 (15%) said yes, it would pose problems but they would not influence curriculum decisions; and 5 (10%) said yes, controversy could make it more difficult to add more evolution content to the curriculum.

**Figure 1:**
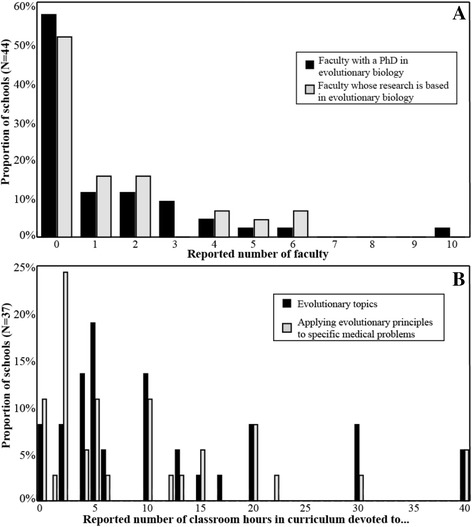
**Faculty and time for teaching evolutionary medicine. A**: Evolutionary biology expertise among North American medical school faculty in 2013 **B**: Reported time spent teaching evolution in North American medical school curricula in 2013. One school that reported 102 hours is not shown here.

The number of faculty at the medical school and hours in the curriculum related to evolution are shown in Figures 1A and B, respectively. The median (IQR) number of faculty with a PhD in evolutionary biology was 0 (0, 2), with 57% (25/44) reporting none. Forty-nine percent (22/45) reported having at least one faculty whose research is based on evolutionary biology, with a median of 0 (0, 2). Responding schools reported a median of 6 (4, 16) hours allocated to teaching specific topics in evolutionary biology and 5 (2, 15) hours devoted to teaching applications of evolutionary principles to specific medical problems. One school claimed to devote 102 hours to teaching how evolution applies to specific medical problems.

### Importance and coverage of topics in evolutionary medicine

**Figure 2:**
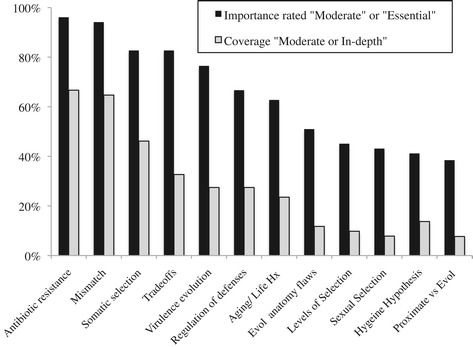
**Rated importance and coverage of evolutionary medicine topics by fifty-one curriculum deans of North American medical schools.**

Figure 2 illustrates the proportion of schools that rated topics as at least *moderately important* and *moderately covered*. The three evolutionary topics most covered were antibiotic resistance (how it is shaped by natural selection), mismatch (how our bodies are ill-adapted to modern environments and how this increases the risk of chronic disease), and somatic selection (how diversity and competition among cells within a tumor influences growth and treatment outcome). The overall mean rating of all 12 topics’ importance at a medical school was highly correlated with the school’s mean reported coverage (r_s_ = 0.76, *P* < 0.01).

### Comparison to 2003 survey results

In comparison to the 2003 survey, we found an increase in the proportion of schools in 2013 with faculty trained in evolutionary biology, devoting at least some time to teaching evolution, and reporting that adding evolutionary content could arouse controversy (Table 2). More schools reported teaching coverage for all nine of the evolution topics asked about in both survey; mean (SD) increase of 44% (26%). However, if coverage of topics is restricted to “moderately” or “in depth” (excluding a response of “covered briefly,” the mean [SD] increase in coverage of a topic drops to 6% [24%].

**Table 2:** **The change in evolutionary medicine resources and instruction over 10 years in North American medical schools**

	2003	2013	Difference
Having any evolutionary biologists on the faculty	16%	43%^a^	+27%
Devoting any curriculum hours to teaching evolution	80%	97%^b^	+17%
Reporting that adding evolutionary content to the curriculum would arouse controversy	11%	48%^c^	+38%
% Reporting coverage of…^d^			
Antibiotic resistance	94%	98%^e^	+4%
Environmental mismatch	30%	94%^e^	+64%
Tradeoffs	26%	90%^e^	+74%
Pathogen virulence	83%	88%^e^	+5%
Aging/Life-history theory	19%	82%^f^	+63%
Defense regulation	20%	80%^e^	+60%
Levels of selection	51%	70%^f^	+19%
Anatomical flaws from path dependence	17%	67%^e^	+50%
Proximate vs. evolutionary explanations of disease	5%	57%^e^	+52%

^a^19/44. ^b^36/37. ^c^23/48. ^d^Percentages for 2003 results include topics reported as *covered* (vs. *not covered*). Percentages for 2013 results include topics reported as *covered briefly*, *covered moderately*, and *covered in depth* (vs. *not covered*). ^e^Denominator = 51. ^f^Denominator = 50.

### Factors influencing coverage of evolution in medical school curricula

A school’s global rating of evolution coverage was correlated with the number of hours devoted to these topics (r_s_ = 0.59, *P* < 0.01), hours spent teaching how to apply evolutionary thinking to specific medical problems (r_s_ = 0.46, *P* < 0.01), and the number of faculty whose research is based in evolutionary biology (r_s_ = 0.38, *P* = 0.02). Having an evolutionary medicine student interest group was associated with higher coverage (t = 2.1, *P* = 0.04). The association between coverage and the number of faculty with a PhD in evolutionary biology at the medical school was moderate and not significant (r_s_ = 0.29, *P* = 0.07). Global coverage of evolutionary medicine topics was not associated with any other school characteristics measured, including country, region of country, source of funding, NIH ranking, priority of research, voting record in the 2012 presidential election, or the anticipated controversy from adding evolution to the school’s curriculum.

### Qualitative responses

Twenty-two of 60 participants responded to open-ended questions. Their 33 comments fell into two major themes- barriers (21) and benefits (12). Barriers were categorized as limited resources (faculty, training, educational materials, 8 comments); limited time (congested curricula, 5 comments); low reported importance (importance of evolutionary biology not evident, 5 comments); and potential controversy (religious opposition, 3 comments). Benefits were categorized as foundational (evolutionary biology is essential to learning and understanding medicine, 8 comments) and applicable (evolutionary biology improves a physician’s ability to treat patients, 4 comments). Limited resources were the most commonly cited barrier; for example, one noted, “lack of faculty expertise” as an obstacle. Curriculum deans varied in how important they considered evolution to be for future physicians. One dean claimed that “Excellent medicine can be practiced without any understanding of evolutionary biology,” while another respondent declared, “The [evolutionary] framework becomes useful (and immediately obvious) as a lens through which to understand and link numerous concepts in medicine.”

### Teaching resources

**Figure 3:**
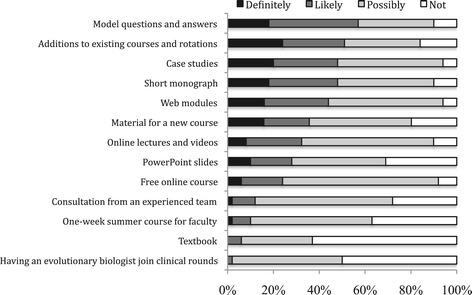
**Rated usefulness of resources for teaching evolution by fifty curriculum deans of North American medical schools.**

Ratings of potential usefulness for 13 teaching resources are reported in Figure 3. The three resources that curriculum deans reported would be most useful were model test questions and answers with explanations, model curricula for adding evolution content to existing courses and rotations, and case studies with facilitator guides. Resources viewed as least useful were having an evolutionary biologist on rounds, a textbook, and a summer course for faculty.

## Discussion

The 60 responses from busy deans that provide the data for this report appear to be an unbiased sample. Compared to a similar study in 2003 [29], this survey of North American medical schools revealed that the proportion of medical schools reporting coverage of key evolutionary medicine concepts in their curriculum has increased over 10 years. Although evolutionary content has increased, large gaps remain between the perceived importance of core evolutionary topics and the extent of their incorporation into medical school curricula. The key barriers to teaching evolution in medical school are few evolution scientists on the faculty, a lack of educational resources, and insufficient time in the packed curriculum. In addition, a few schools anticipated that adding more evolutionary content to their curriculum would arouse controversy.

When comparing results from the survey of medical school deans a decade ago [29], we found substantial expansion of teaching key evolutionary topics. To avoid confusion over terminology reported by responders to the previous survey, our survey defined the evolutionary concepts and gave a sample learning objective. This change in methodology may be more responsible for the large range in increased coverage (4 to 74%) than actual changes in curricula. The number of medical schools reporting at least one faculty member trained in evolutionary biology more than doubled- from 16% to 43%. However, since the surveys do not involve the same schools, these trends should be interpreted cautiously.

Despite calls from the AAMC and others for a strong undergraduate education in evolutionary biology for future health professionals [31], we found that less than half (44%) of schools reported valuing or recommending an undergraduate course in evolutionary biology for admission. Nonetheless, interest in such courses among applicants is likely to increase, because the Medical College Admission Test (MCAT), administered by the American Association of Medical Colleges (AAMC), will begin incorporating more questions on evolutionary biology in 2015. This change in the MCAT will help meet a recommendation presented in the 2009 *Scientific Foundations for Future Physicians* report from a committee commissioned by the AAMC and the Howard Hughes Medical Institute [32].

The number of faculty whose research is based in evolutionary biology was associated with the school’s current coverage of evolutionary topics. These individuals may be directly responsible for higher coverage at their institutions; a few role models have reported a warm reception from medical students given an opportunity to learn evolutionary biology [3,28,30]. Medical school curricula are already overflowing with content, so it is not surprising to find limited time commonly reported as a barrier to adding evolutionary content. Deans reported little interest in new courses, but considerable interest in strategies for infusing evolution into existing curricula. The three most popular teaching resources were model questions and answers with explanations, case studies, and additions to existing courses and rotations. Few were interested in having an evolutionary biologist join clinical rounds, although feedback from such experiences at the University of California Los Angeles David Geffen School of Medicine, New York University School of Medicine, and the University of Michigan School of Medicine have been positive [personal communication from Stephen C. Stearns, Randolph M. Nesse, and Andrew F. Read].

A 2013 poll found 1 in 3 American adults rejects evolution as an explanation for human origin [33], so it is not surprising that almost half (48%) of responders anticipated igniting at least some controversy at their medical school if they added more content about evolution. One in 10 reported that controversy could make it more difficult to add more evolution at the curriculum. A survey of medical students in the U.K. found that religious beliefs were the most commonly cited reason for rejecting evolution [28]. We found that medical school is not immune to the social forces that stymie teaching biological science at other education levels [34-36].

Our survey has limitations. First, the sample may be biased by self-selection. If responders were more interested in evolution than those that did not respond, then our results would be overly optimistic about the status of evolutionary education in North American medical schools. The low response rate in itself may indicate a low priority of the subject. Secondly, we did not directly measure the evolutionary content of medical curricula. Our data reflect the reports of curriculum deans’ offices and questions may have been interpreted differently among responders. For example, our questions regarding time in the curriculum intended to ask about total time throughout the four years of medical school. Future surveys should be more specific.

Research on evolutionary biology in medical education could advance in several ways. First, sponsorship of a similar study by the AAMC or another major medical education organization would likely improve the response rate. A more detailed review and coding of actual curricular materials, rather than potentially subjective reports, would deepen understanding of the curricular emphasis on evolution in North American medical schools. Controlled studies would enable comparisons of how medical practice and research are different in physicians who learn evolutionary biology compared to those who do not. However, this is a high bar to set, one not required for teaching biochemistry, physics, or embryology. Evolutionary biology is a basic science whose contributions to medicine are still being explored [1]. Organic chemistry is required for medical school admission because it arms students with principles for understanding how molecules react and interact to create structure and function, not because it makes a physician better in a direct way. Analogously, evolutionary biology gives students the tools to understand why our bodies seem so exquisitely designed, yet susceptible to innumerable maladies.

## Conclusion

We live in a rapidly changing world with emerging infectious diseases, evasive cancer cells, and novel environments. Teaching medical students about our evolutionary legacy and the biological forces that shaped our past will help them to be better prepared for our future. And the interest expressed by curriculum deans indicates that opportunities for infusing evolutionary thinking into medical education are within reach.

## Additional file

Additional file 1:
**Survey packet including cover letter and questionnaire.**
