# Supplementary material for: The status of evolutionary medicine education in North American medical schools
Source: BMC Med Educ. 2015 Mar 8;15:38. doi: 10.1186/s12909-015-0322-5 (PMC4355969; doi:10.1186/s12909-015-0322-5)
Supplement: Additional file 1: — Survey packet including cover letter and questionnaire. [file 12909_2015_322_MOESM1_ESM.pdf]

Survey:
The Role of Evolutionary Concepts in Medical Education

Survey of Medical School Curriculum Deans on
The Role of Evolutionary Concepts in Medical Education

Dear Dean,

This letter is to request your help completing a brief questionnaire for a study on the teaching of evolutionary principles in North American medical schools, and what resources Deans think would be most useful to incorporate these principles into medical education. It should take no more than 10 minutes to complete, and a stamped, addressed envelope is provided.

The study is sponsored by the National Science Foundation (NSF), and by the National Evolutionary Synthesis Center (NESCent) and it is led by Anila Asghar, EdD at McGill University, and Mark Schwartz, MD at NESCent and New York University. It builds on the 2009 HHMI/AAMC learning objectives for evolution[1] and a 2010 PNAS article on how to implement them.[2]

Harvey Fineberg, President of the Institute of Medicine and Chair of the Advisory Committee for our NESCent initiative, strongly supports this effort, saying:

> ***It will be critical to learn from initiatives already underway in many schools, and I personally urge all deans to provide their input to this important survey.***

The confidentiality and privacy of our participants is protected. Your name and institutional information will not be disclosed in our research reports and published articles. Your participation is entirely voluntary. We do not foresee any potential risks or discomfort as a result of participating in this study. Your questionnaire has been assigned a unique code linked to your institution to allow us to follow up with non-responders in order to achieve an adequate response rate. A reference list linking the codes to the institutions will be kept in a separate, safe, and secure location that only Dr. Asghar will have access to. Please contact Dr. Asghar if you wish you to withdraw your survey data.

We are grateful for your help, and would be happy to provide you with results from the survey.

With thanks and best wishes,

Anila Asghar, Ed.D. (anila.asghar@mcgill.ca)
Associate Director, Evolution Education Research Center
Department of Integrated Studies in Education
McGill University

Mark D. Schwartz, MD (Mark.Schwartz3@va.gov)
Vice-Chair, Department of Population Health
New York University School of Medicine

---

[1] Scientific Foundations for Future Physicians: Report of the AAMC-HHMI Committee, 2009. http://www.hhmi.org/grants/pdf/08-209_AAMC-HHMI_report.pdf
[2] Nesse RN, et al. Making Evolutionary Biology a Basic Science for Medicine. Proc Nat Acad Sci 2010;107:1800-1807.

The McGill University maintains an Ethics Review Board for studies using human subjects. Any complaints or problems concerning any research project may, and should, be reported to the board at 514-398-6193 or lynda.mcneil@mcgill.ca

Section A

At this medical school, the priority of training future researchers is

a. Low
b. Medium
c. High
d. Very High

At this medical school, an undergraduate course in evolutionary biology is

a. Not considered in admissions or preparation guidelines
b. Valued, but not formally recommended
c. Recommended
d. Required as a prerequisite

Number of medical faculty FTE with a PhD in evolutionary biology (approx.) ________

Number of medical faculty FTE whose research is based in evolutionary biology (approx.) ________

Section B

Each of the items below consists of **a topic**, a related evolutionary **principle**, and an example of a **learning objective**. Please indicate your personal assessment of the importance of teaching the topic/principle, and the amount of coverage of the topic in the current curriculum at your school, by circling one response on each of the two italicized lines at the end of each item.

1. **<u>Maladaptive aspects of anatomy:</u>** Bodies are different from machines in that they are shaped by incremental changes, so fundamental redesign is not possible. Use this principle to explain why bodies are vulnerable to volvulus, dystocia, and choking.

Importance	*a) Not important*	*b) Minor importance*	*c) Moderate importance*	*d) Essential*
Current coverage:	*a) Not covered*	*b) Covered briefly*	*c) Covered moderately*	*d) Covered in depth*

2. **<u>How natural selection shapes defenses:</u>** Many medical complaints are about aversive but protective defenses such as pain, cough, vomiting, diarrhea, and anxiety. Explain how to distinguish symptoms that are defenses from those that arise from bodily defects, and the importance of making this distinction.

Importance	*a) Not important*	*b) Minor importance*	*c) Moderate importance*	*d) Essential*
Current coverage:	*a) Not covered*	*b) Covered briefly*	*c) Covered moderately*	*d) Covered in depth*

3. **How selection shapes pathogen virulence:** Virulence does not decrease with long host-pathogen association, it evolves to whatever level maximizes transmission. Use this principle to explain why pathogens transmitted by vectors tend to be more virulent than those transmitted by direct person-to-person contact in the community.

Importance	*a) Not important*	*b) Minor importance*	*c) Moderate importance*	*d) Essential*
Current coverage:	*a) Not covered*	*b) Covered briefly*	*c) Covered moderately*	*d) Covered in depth*

4. **Proximate versus evolutionary explanation:** Every biological trait needs an evolutionary explanation of its origins and functions as well as a proximate explanation of its mechanisms and ontogeny. Explain why diseases themselves do not have evolutionary explanations, and strategies for finding evolutionary explanations for traits that make bodies vulnerable to disease.

Importance	*a) Not important*	*b) Minor importance*	*c) Moderate importance*	*d) Essential*
Current coverage:	*a) Not covered*	*b) Covered briefly*	*c) Covered moderately*	*d) Covered in depth*

5. **Somatic selection:** Selection among cells in a tumor explains tumor growth and diversity. Explain the implications for cancer therapy.

Importance	*a) Not important*	*b) Minor importance*	*c) Moderate importance*	*d) Essential*
Current coverage:	*a) Not covered*	*b) Covered briefly*	*c) Covered moderately*	*d) Covered in depth*

6. **Trade-offs:** The optimality of every biological trait is limited by trade-offs. Explain how the capacity for growth and tissue repair leave bodies susceptible to cancer.

Importance	*a) Not important*	*b) Minor importance*	*c) Moderate importance*	*d) Essential*
Current coverage:	*a) Not covered*	*b) Covered briefly*	*c) Covered moderately*	*d) Covered in depth*

7. **Mismatch between our bodies and modern environments:** The majority of chronic disease in modern societies arises from aspects of modern environments for which our bodies are ill-prepared. Use this to explain the prevalence of Type 2 diabetes mellitus and atherosclerosis.

Importance	*a) Not important*	*b) Minor importance*	*c) Moderate importance*	*d) Essential*
Current coverage:	*a) Not covered*	*b) Covered briefly*	*c) Covered moderately*	*d) Covered in depth*

8. **<u>Hygiene hypothesis:</u>** As a population's exposure to helminths decreases, the incidence of asthma and inflammatory bowel disease increases. Offer a likely evolutionary explanation for this finding.

Importance	*a) Not important*	*b) Minor importance*	*c) Moderate importance*	*d) Essential*
Current coverage:	*a) Not covered*	*b) Covered briefly*	*c) Covered moderately*	*d) Covered in depth*

9. **<u>Senescence/aging:</u>** Natural selection has not eliminated some alleles that cause aging because they do not cause deleterious effects during the lifespan in the wild, and it preserves others because they give advantages in youth (antagonistic pleiotropy). What are the implications for understanding aging and age-related diseases?

Importance	*a) Not important*	*b) Minor importance*	*c) Moderate importance*	*d) Essential*
Current coverage:	*a) Not covered*	*b) Covered briefly*	*c) Covered moderately*	*d) Covered in depth*

10. **<u>Sexual selection</u>:** Traits that increase mating success are often selected for, even if they decrease individual health and longevity. How can this principle explain sex differences in mortality rates?

Importance	*a) Not important*	*b) Minor importance*	*c) Moderate importance*	*d) Essential*
Current coverage:	*a) Not covered*	*b) Covered briefly*	*c) Covered moderately*	*d) Covered in depth*

11. **<u>Antibiotic resistance</u>:** Natural selection quickly shapes antibiotic resistance. Explain the evolutionary principles that can help physicians decide whether it is wise to cycle among different first choice antibiotics in a hospital.

Importance	*a) Not important*	*b) Minor importance*	*c) Moderate importance*	*d) Essential*
Current coverage:	*a) Not covered*	*b) Covered briefly*	*c) Covered moderately*	*d) Covered in depth*

12. **<u>Levels of selection</u>:** Selection is far stronger at the levels of genes and individuals than it is for groups or species. Explain how this principle applies to the hypothesis that mutation rates are shaped by selection to speed evolution of a species when environments change.

Importance	*a) Not important*	*b) Minor importance*	*c) Moderate importance*	*d) Essential*
Current coverage:	*a) Not covered*	*b) Covered briefly*	*c) Covered moderately*	*d) Covered in depth*

Section C

1 About how many classroom hours in the curriculum are devoted to teaching specific topics in evolutionary biology such as those listed above? ____________ hours total

2 About how many classroom hours in the curriculum are devoted to teaching applications of evolutionary principles to specific medical problems? (e.g. why the birth canal is narrow, how to trace pathogen phylogenies, tradeoffs that make some resistant bacteria less competitive when antibiotics are not present, why selection does not eliminate alleles that cause aging) ____________ hours total

3 Does your medical school have a student club or interest group focused on evolution and medicine? 1. Yes 2. No

4 Would adding more content about evolution to the curriculum at your school arouse controversy among students, faculty, legislators, or donors?

1. No
2. Yes, but it would not pose any problems
3. Yes, and it would pose problems, but they would not influence curriculum decisions
4. Yes, controversy could make it difficult to add more evolution content to the curriculum

Comments about possible effects of controversy at your school

____

____

____

Section D

How useful would each of the following resources be at your school?

For each item, please circle the item that best reflects your assessment

1. Model curricula for brief medical school courses on evolutionary applications in medicine

   a) Not useful *b) Possibly useful* *c) Likely useful* *d) Definitely useful*

2. Model curricula for ways to add evolution content to existing courses and clinical rotations

   a) Not useful *b) Possibly useful* *c) Likely useful* *d) Definitely useful*

3. Case studies with facilitator guides that infuse evolutionary principles into basic or clinical science discussions

   a) Not useful *b) Possibly useful* *c) Likely useful* *d) Definitely useful*

4. A textbook covering evolutionary principles and their application in medicine

   a) Not useful *b) Possibly useful* *c) Likely useful* *d) Definitely useful*

5. A free online course on evolution and medicine

   a) Not useful *b) Possibly useful* *c) Likely useful* *d) Definitely useful*

6. A short monograph summarizing 50 things every physician needs to know about evolution

   a) Not useful *b) Possibly useful* *c) Likely useful* *d) Definitely useful*

7. Online lectures/videos on topics relevant to evolution and medicine

   a) Not useful *b) Possibly useful* *c) Likely useful* *d) Definitely useful*

8. PowerPoint slide sets for teaching

   a) Not useful *b) Possibly useful* *c) Likely useful* *d) Definitely useful*

9. Web modules (5-20 minutes) about specific medical applications of evolutionary principles that can integrate with existing classroom lectures

   a) Not useful *b) Possibly useful* *c) Likely useful* *d) Definitely useful*

10. Model test questions and answers with explanations

    a) Not useful *b) Possibly useful* *c) Likely useful* *d) Definitely useful*

11. Having an evolutionary biologist join clinical rounds on occasion.

    a) Not useful *b) Possibly useful* *c) Likely useful* *d) Definitely useful*

12. One week intensive summer course for faculty to learn evolutionary applications in medicine

    a) Not useful *b) Possibly useful* *c) Likely useful* *d) Definitely useful*

13. A curriculum consultation with a biologist/physician team experienced with implementing evolutionary components in medical curricula.

    a) Not useful *b) Possibly useful* *c) Likely useful* *d) Definitely useful*

Section E

We would especially value any additional thoughts you would be willing to share about:

1. How you see evolutionary principles being useful in medicine (or not):
____
____
____
____
____

2. Your thoughts about why evolutionary biology has not been fully applied in medicine:
____
____
____
____
____

3. Any suggestions you have for what resources would be helpful to better infuse evolutionary thinking into medical education:
____
____
____
____
____

If you would like to receive the aggregate results of this survey and information about new resources, please provide your email address here: ____________________________________

Space for further comments:

We thank you very much for your time and responses.

Please put this survey in the preaddressed stamped envelope and put it in the mail.

If the envelope is missing, the address is:

Dr. Anila Asghar
Evolution Education Research Center
McGill University
3700 McTavish Street - Rom 364
Montreal, Quebec
H3A 1Y2
Canada

Dr. Anila Asghar
Centre de recherche en éducation de l’évolution
Université McGill
3700 rue McTavish – Salle 364
Montréal, Québec
H3A 1Y2
Canada
